# Obituary

**Published:** 1850-01

**Authors:** 


### ARTICLE VIII.

### OBITUARY.

Just as we go to press we hear of the death, on the 3rd instant, of Dr. Samuel B. Woodward, of Northampton, Mass., late Superintendent of the Massachusetts State Lunatic Hospital, at Worcester. Dr. Woodward for thirteen years conducted that celebrated institution, with distinguished ability and success. His opinions upon subjects connected with insanity have had great influence throughout the United States.

